# Yield-stress fluids in porous media: a comparison of viscoplastic and elastoviscoplastic flows

**DOI:** 10.1007/s11012-019-01010-6

**Published:** 2019-07-17

**Authors:** Emad Chaparian, Daulet Izbassarov, Francesco De Vita, Luca Brandt, Outi Tammisola

**Affiliations:** Linné FLOW Centre and SeRC, Mechanics Department, Royal Institute of Technology (KTH), Stockholm, Sweden

**Keywords:** Porous media, Yield-stress fluid, Viscoplastic fluid, Elastoviscoplastic fluid

## Abstract

A numerical and theoretical study of yield-stress fluid flows in two types of model porous media is presented. We focus on viscoplastic and elastoviscoplastic flows to reveal some differences and similarities between these two classes of flows. Small elastic effects increase the pressure drop and also the size of unyielded regions in the flow which is the consequence of different stress solutions compare to viscoplastic flows. Yet, the velocity fields in the viscoplastic and elastoviscoplastic flows are comparable for small elastic effects. By increasing the yield stress, the difference in the pressure drops between the two classes of flows becomes smaller and smaller for both considered geometries. When the elastic effects increase, the elastoviscoplastic flow becomes time-dependent and some oscillations in the flow can be observed. Focusing on the regime of very large yield stress effects in the viscoplastic flow, we address in detail the interesting limit of ‘flow/no flow’: yield-stress fluids can resist small imposed pressure gradients and remain quiescent. The critical pressure gradient which should be exceeded to guarantee a continuous flow in the porous media will be reported. Finally, we propose a theoretical framework for studying the ‘yield limit’ in the porous media.

## Introduction

Non-Newtonian fluid flows in porous media are of great practical importance for numerous industries such as filtration and polymer extrusion. However, several important effects remain to be explored in these flows; from inhomogeneity associated with the structure of the medium to the complexity of the behavior of the fluid passing through the solid matrix. For instance, non-Newtonian fluids will break the validity of Darcy’s law [13] (because of intrinsic non-linearity in the constitutive equations) and so have been investigated numerously [42]. The non-linear behavior of the fluid can have different origins. It may stem from the elastic behavior of the fluid or more complex phenomena such as jamming in suspensions passing through porous media [1].

For instance, De et al. [17, 18], very recently, studied the viscoelastic flow over two model porous media at the highly elastic regime. The effects of moderate and high inertia were also investigated in viscoelastic fluid flows through the arrays of cylinders [33, 45]. In this paper, we address the yield-stress fluid flows in porous media to continue and connect to the findings of our previous study [16].

A yield-stress fluid exhibits interesting features which to some extent combines “solid-like” and “fluid-like” behaviors: below a certain level of the imposed stress, it can be described as a solid, whereas beyond that critical value, the imposed stress can make it flow like a fluid. Hence, the term “yield stress” is used to mark the threshold of the imposed stress that should be exceeded to make these type of materials flow. Due to this unique characteristic, yield-stress fluid flows in porous media is not a trivial problem to study [42]. However, because of substantial engineering applications of yield-stress fluid flows in porous media (e.g., oil recovery) both experimental and computational studies exist in the literature.

Chevalier et al. [10] experimentally studied the flow of a Herschel-Bulkley fluid through confined packings of glass beads and proposed a semi-empirical model for the pressure drop versus the flow rate. Yield-stress fluid flow inside packed-bed models of porous media was investigated experimentally by Chevalier et al. [11] and also numerically by Bleyer and Coussot [2]. In another interesting work by Talon and Bauer [44], the Lattice Boltzmann Method (LBM) was used to study Bingham fluid flow in an stochastically reconstructed porous media. These authors showed that due to the yield stress, channelization can happen: the fluid will flow only in self-selected paths depending on the imposed driving pressure difference. Another related topic is the flow along uneven channels. In the context of yield-stress fluids, Roustaei et al. [35–38] investigated in detail the yield-stress fluid flow inside fractures and washouts for different flow conditions from Stokes to inertial flows. They have shown that large-amplitude variations in the duct walls lead to the formation of static unyielded zones (termed as ‘fouling layers’) adjacent to the walls. These formations will restrict the usage of the lubrication approach (which is based on a constant pressure gradient along the channel length) to study yield-stress fluid flows along wavy channels. It should be mentioned that all the above studies are limited to the viscoplastic (VP) fluids which can be categorized as ‘simple’ yield-stress fluids.

Apart from ‘simple’ yield-stress fluids, different attempts have been made to categorize more complex yield-stress fluids from different perspectives (e.g., see [31]). Furthermore, a large number of rheological models have been proposed to describe the behavior of ‘non-simple’ yield stress fluids from thixotropy effects [12, 26] to elasticity effects [39, 40]. More complex models such as structural-based models aim to combine thixotopy, elasticity, and plasticity [14] (an overview of these type of models can be found in Ref. [15]). Very recently, based on isotropic/kinematic hardening, Dimitriou and McKinley [19] have built up a framework for predicting the multiplex (thixo-elastoviscoplastic) behavior of waxy crude oil which is termed as the IKH model. In a similar context, Fraggedakis et al. [23] examined four different rheological models of elastoviscoplastic (EVP) fluids with large amplitude oscillatory shear (LAOS) database and have verified that Saramito’s model [39] is a reliable representation of this type of fluids. Moreover, it has been shown that Saramito model, in spite of its simplicity compare to the other EVP models (ignoring more complex behaviours such as shear-thinning effects, thixotropy, and residual stresses), can capture complex fascinating behaviors such as the negative wake in particle sedimentation [22] through Carbopol gel and the sculpture of a foam flowing over an obstacle [9]. So in the present study, we will rely on Saramito’s model to represent the dynamics of an EVP fluid. In this model, before yielding, the material behaves as a viscoelastic solid, whereas after yielding, its behavior is described by a viscoelastic (Oldroyd-B) fluid.

In this work, we solve the full Cauchy equations governing the inertial flows of a viscoplastic fluid (Bingham model) and elastoviscoplastic fluid (Saramito model). The focus is on the motion of a yield-stress fluid through a solid matrix (model porous media). Our paper has two main objectives. First, we use the numerical results to investigate the yield-stress fluid flows in complex geometries such as porous media and find general addresses about pressure drop and ‘yield limit’. Second, we aim to analogously explore the difference/similarity of VP and EVP flows in porous media. One of the longer term objectives would be understanding of how elasticity effects (which have been neglected usually in the literature) change the flow of a yield-stress fluid in different physical problems.

The outline of the paper is as follows. In Sect. 2, we present the problem statement. In the results section (Sect. 3), we will consider the VP and EVP flows in the model porous media. Firstly, the general features of the flows will be compared. Secondly, the pressure drop for different geometries and broad range of effective parameters will be reported. Finally, the limit of ‘flow/no flow’ will be investigated in detail and a theoretical framework will be proposed for a deeper understanding of this crucial limit and the minimal pressure difference that should be exceeded to move the fluid. The paper closes with a brief summary and discussion section.

## Problem statement

**Fig. 1:**
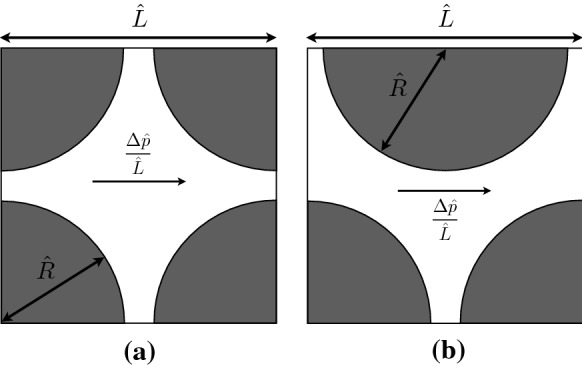
Schematic of the model porous media: **a** regular geometry, **b** staggered geometry. The whole domain (inside of the square box $\hat{L} \times \hat{L}$) is $\varOmega$, while the solid topologies (which are shaded in gray) are *X*. The center of the coordinate system is at the center of the inlet gap and $\hat{L}=2.25\hat{R}$

Porous media exhibit inhomogeneity due to the randomness of their structure. However, a common way is to model the medium as arrays of cylinders [16, 17, 29]. In the present study, two model geometries of 2D porous media are considered as illustrated in Fig. 1: regular and staggered structures. We perform the computations in only one cell with periodic boundary condition at the inlet and the outlet. As it can be seen, the solid matrix has been drafted so the porosity is conserved in both geometries (i.e., it is equal to $1-\frac{\pi }{2.25^2}$). The incompressible flow is driven from left to right by a ‘mean’ pressure gradient, $\varDelta \hat{p}/\hat{L}$.

In this study we consider two different rheological models for the behavior of yield-stress fluids. For the VP fluid we use the classical Bingham model, while for the EVP fluid, the Saramito model [39] (without shear-thinning effects) has been used.

To scale the governing and constitutive equations, we introduce the following non-dimensional numbers,

$$\begin{aligned} {\mathcal{R}}e = \frac{\hat{\rho } \hat{U} \hat{R}}{\hat{\mu }},~~{\mathcal{B}} = \frac{{\hat{\tau }}_Y \hat{R}}{\hat{\mu } \hat{U}},~~\text{ and }~~{\mathcal{W}}i = \frac{\hat{\lambda } \hat{U}}{\hat{R}}, \end{aligned}$$

the Reynolds, Bingham and Weissenberg numbers, respectively. The ‘hat’ sign ($\hat{\cdot }$) indicates dimensional parameters. Here, $\hat{R}$ is the radius of the solid topologies (see Fig. 1), $\hat{\rho }$ is the density of the fluid, $\hat{\tau }_Y$ the yield stress of the fluid, and $\hat{U}$ is the mean velocity at the inlet. Moreover, $\hat{\lambda }$ is the relaxation time of the EVP fluid ($\hat{\lambda }=0$ for the VP fluid) and $\hat{\mu }$ is the characteristic viscosity which takes different interpretations based on the rheology of the fluid. For the VP fluid, the *plastic* viscosity ($\hat{\mu }_p$) is the only source of viscous dissipation and hence, $\hat{\mu } = \hat{\mu }_p$. For the EVP fluids, on the other hand, there are two viscous parameters: $\hat{\mu }_1$ and $\hat{\mu }_2$, usually called *solvent* and *polymeric* viscosities. Hence, for the EVP fluid we use: $\hat{\mu } = \hat{\mu }_1 + \hat{\mu }_2$.

Therefore, by scaling the velocity vector ($\hat{\varvec{u}} = (\hat{u},\hat{v})$) with $\hat{U}$, pressure ($\hat{p}$) with $\hat{\rho } \hat{U}^2$, and the extra stress tensor ($\hat{\varvec{\tau }}$ which is deviatoric for VP fluid but not necessarily for the EVP fluid) with the characteristic viscous stress, $\hat{\mu } \hat{U}/\hat{R}$, the governing and constitutive equations take the form,

1 $$\begin{aligned} {\mathcal{R}}e \frac{\text{ D } \varvec{u}}{\text{ D } t}= - {\mathcal{R}}e~ \nabla p + \varvec{\nabla } \cdot \varvec{\tau } + \beta ~ \varvec{\nabla } \cdot \dot{\varvec{\gamma }}, \end{aligned}$$

and,

2 $$\begin{aligned} {\mathcal{W}}i ~\overset{\triangledown }{\varvec{\tau }} + \left( 1 - \frac{{\mathcal{B}}}{\Vert \varvec{\tau }_d \Vert } \right) _+ \varvec{\tau }= \left( 1 - \beta \right) \dot{\varvec{\gamma }}, \end{aligned}$$

respectively. In the above equations, $\beta$ represents $\hat{\mu }_2 / \hat{\mu }$, $\dot{\varvec{\gamma }}$ is the rate of strain tensor, $\overset{\triangledown }{\varvec{\tau }}$ the upper-convected derivative of the extra stress tensor [39], $\varvec{\tau }_d$ the deviatoric part of the extra stress tensor, and,

$$\begin{aligned} \left( \varGamma \right) _+ = \left\{ \begin{array}{ll} \varGamma ~~&{} \text{ iff }~ \varGamma > 0, \\ 0 ~~&{} \text{ iff }~ \varGamma \leqslant 0. \end{array} \right. \end{aligned}$$

Please note that expression (2) holds for both a Saramito fluid (EVP fluid) and a Bingham fluid (VP fluid) provided that for a VP fluid, both ${\mathcal{W}}i$ and $\beta$ are equal to zero and also $\varvec{\tau }_d = \varvec{\tau }$ [22]. In this case (${\mathcal{W}}i = 0 ~ \& ~ \beta = 0$), expression (2) can be rewritten as,

3 $$\begin{aligned} \left\{ \begin{array}{ll} \varvec{\tau } = \left( 1 + \displaystyle {\frac{{\mathcal{B}}}{\Vert \dot{\varvec{\gamma }}\Vert }} \right) \dot{\varvec{\gamma }} ~~&{} \text{ iff }~ \Vert \varvec{\tau } \Vert > {\mathcal{B}}, \\ \dot{\varvec{\gamma }} = 0 ~~&{} \text{ iff }~ \Vert \varvec{\tau } \Vert \leqslant {\mathcal{B}}, \end{array} \right. \end{aligned}$$

which is the classical way of representing the Bingham fluid. Please note that $\Vert \cdot \Vert$ is the norm associated with the tensor inner product ($\varvec{c} \varvec{:} \varvec{d} = \frac{1}{2} \sum _{ij} c_{ij} d_{ij}$), e.g., $\Vert \varvec{f} \Vert = (\varvec{f} \varvec{:} \varvec{f})^{\frac{1}{2}}$.

In summary, the problem at hand is governed by four parameters: $\left( {\mathcal{R}}e, {\mathcal{B}}, {\mathcal{W}}i, \beta \right)$. In what follows, when all four parameters are non-zero, we are looking at the EVP problem, while when ${\mathcal{W}}i$ and $\beta$ are zero, we consider the problem associated with the VP fluid.

A brief introduction of the numerical method is provided in the “Appendix A”. Regarding the boundary conditions, a periodic BC has been imposed at the inlet and outlet, while the no-slip ($\varvec{u}=0$) on the surfaces of the solid cylinders is enforced. For the horizontal faces, the symmetry BC has been imposed.

## Results

We now examine the behavior of the flow and report different quantities (e.g., pressure drop) in this section. To focus on the effect of fluid rheology, the Reynolds number has been fixed at 0.8 in the whole study. Moreover, the effect of ${\mathcal{W}}i$ in the EVP flow in the porous media has been investigated in our previous study [16]. So here the emphasis is on weakly elastic flows (i.e., low ${\mathcal{W}}i$ numbers) to study the differences and similarities with the VP flows in porous media.

### General comparison of VP and low ${\mathcal{W}}i$ EVP flows

**Fig. 2:**
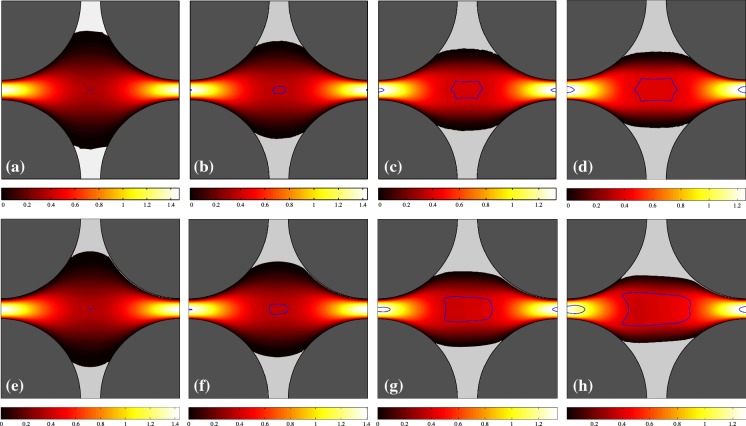
Contour of magnitude of the velocity, $\vert \varvec{u} \vert$, for the regular geometry: panels left to right pertain the simulations with ${\mathcal{B}} = 0.1,~1,~5,~10$. Top panel shows the VP flow while bottom panels are for the EVP flow. In panels (**e**–**h**), the Weissenberg number is 0.01 and $\beta =0.5$. The solid matrix is shaded in dark gray. Blue lines show the moving yield surfaces and static unyielded regions are filled with light gray. (Color figure online)

**Fig. 3:**
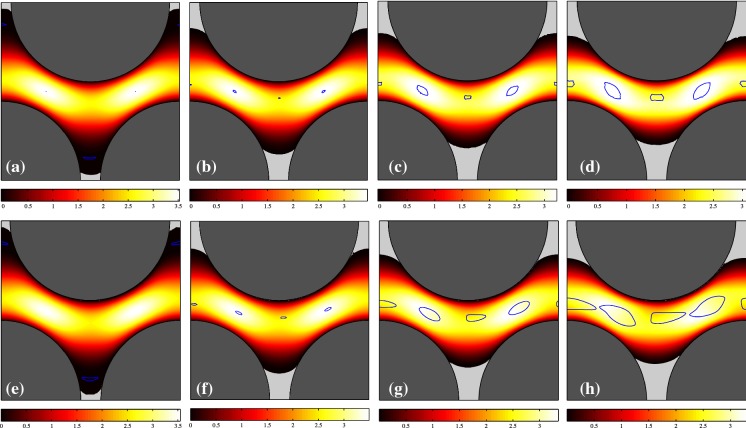
Contour of magnitude of the velocity, $\vert \varvec{u} \vert$, for the staggered geometry: panels left to right pertain the simulations with ${\mathcal{B}} = 0.1,~1,~5,~10$. Top panel shows the VP flow while bottom panels are for the EVP flow. In panels (**e**–**h**), the Weissenberg number is 0.01 and $\beta =0.5$. The solid matrix is shaded in dark gray. Blue lines show the moving yield surfaces and static unyielded regions are filled with light gray. (Color figure online)

In this part, the general features of the VP and EVP flows are examined in the two considered geometries. The Bingham number is varied between 0.1 and 10. The ${\mathcal{W}}i$ and $\beta$ are fixed at 0.01 and 0.5, respectively. Figures 2 and 3 show the contours of the velocity magnitude for ${\mathcal{B}}=(0.1,1,5,10)$ for the regular and staggered geometries. Top panels are the VP flows and bottom panels represent the EVP flow where the static fouled regions are presented in light-gray and the border of the moving unyielded zones (moving yield surfaces) are painted in blue. For a Bingham fluid (on account of the AL method), the unyielded surfaces can be detected either by seeking the $\Vert \dot{\varvec{\gamma }} \Vert =0$ contours or $\Vert \varvec{\tau } \Vert = {\mathcal{B}}$ which are strictly the same (up to numerical error). For an EVP fluid, on the other hand, the unyielded regions are not generally rigid and can resist viscoelastic deformations. Hence to find the unyielded zones in the EVP flows, we have set the criterion $\left( 1 - \frac{{\mathcal{B}}}{\Vert \varvec{\tau }_d \Vert } \right) _+ \leqslant 5\times 10^{-2}$. It should be noted that going to smaller values does not substantially change the shape of the unyielded regions, but by setting $5\times 10^{-2}$ (rather than the absolute zero), the negligibly small wiggles of unyielded zones (which could be the consequence of using staggered mesh in the EVP flow solver) will vanish.

**Fig. 4:**
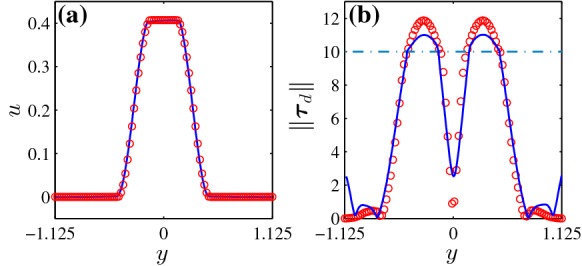
Comparison of VP (red symbols) and EVP (blue lines) solutions at the vertical symmetry line in the middle of the channel for the regular geometry (${\mathcal{B}}=10$): **a** horizontal velocity component versus *y*; **b** second invariant of the deviatoric stress tensor. The green dotted line in the panel (**b**) shows the $\Vert \varvec{\tau }_d \Vert = {\mathcal{B}} = 10$. This figure refers to the flows in panels (d) and (h) of Fig. 2. (Color figure online)

As expected, by increasing the Bingham number, the unyielded zones grow, which is clearly visible from Figs. 2 and 3 for both VP and EVP fluids. By comparing the VP and EVP flows at a same Bingham number, we find out some degree of commonality. However, the moving unyielded regions (blue colored islands in the middle of the channel) are bigger in the EVP flows. This is true for both regular and staggered geometries. Figure 4 compares the velocity and stress distributions in the middle of the channel ($x=1.125$) for the regular geometry in the panels (d) and (h) of Fig. 2. In particular, Fig. 4b shows the second invariant of the deviatoric stress tensor (i.e., von Mises stress) which determines if the fluid is yielded or not. Figure 4 clearly reveals that the velocity fields of VP and EVP flows are nearly identical (and so $\dot{\varvec{\gamma }}$), but stress fields still differ, even though the trends are similar. This is the main reason why the moving unyielded regions are not the same in the VP and EVP flows. Indeed, in the EVP flows, the moving unyielded regions are viscoelastic solids which can resist small strain rates and deform elastically. The von Mises stress of the EVP flow varies more smoothly in the central region of the channel, due to the elastic contributions to the stress. It is worth noting that the computed stress field below the yield stress in any VP flow is only an admissible stress field and generally there is no unique solution for the stress below the yield stress [8].

Finally, it should be mentioned that some right-left asymmetry (with respect to the vertical symmetry line in the middle of the channel) can be observed in the static unyielded regions in the EVP flow, whereas it is not significant in the VP flows because of low inertia. Hence, we can claim that elasticity of the fluid is the main cause of this asymmetry.

### Pressure drop

From a practical view, the pressure difference which drives the flow is of primary importance. For instance, for the VP flow, the energy balance could be written as,

4 $$\begin{aligned} {\mathcal{R}}e~b(\varvec{u},\varvec{u},\varvec{u}) + \frac{1}{2} a(\varvec{u},\varvec{u}) + {\mathcal{B}} j(\varvec{u}) = {\mathcal{R}}e \int _{\varOmega \setminus \bar{X}} \frac{\text{ d } p}{\text{ d } x}~u~\text{ d }A, \end{aligned}$$

where,

$$\begin{aligned}&b(\varvec{u},\varvec{v},\varvec{w}) = \int _{\varOmega \setminus \bar{X}} \varvec{u} \cdot \varvec{\nabla }\varvec{v} \cdot \varvec{w} ~\text{ d }A, \\&a(\varvec{u},\varvec{v}) = \int _{\varOmega \setminus \bar{X}} \dot{\varvec{\gamma }} (\varvec{u}) \varvec{:} \dot{\varvec{\gamma }} (\varvec{v})~\text{ d }A, \end{aligned}$$

and,

$$\begin{aligned} j(\varvec{v}) = \int _{\varOmega \setminus \bar{X}} \Vert \dot{\varvec{\gamma }} (\varvec{v}) \Vert ~\text{ d }A, \end{aligned}$$

are the inertial power functional, the viscous dissipation, and the plastic dissipation, respectively, while the term on the right-hand side (RHS) of Eq. (4) is the body force functional. We can substitute the integral on the RHS by $\frac{\varDelta p}{L} \int _{\varOmega } u~\text{ d }A$, where $\varDelta p / L$ is the ‘mean’ pressure gradient.

**Fig. 5:**
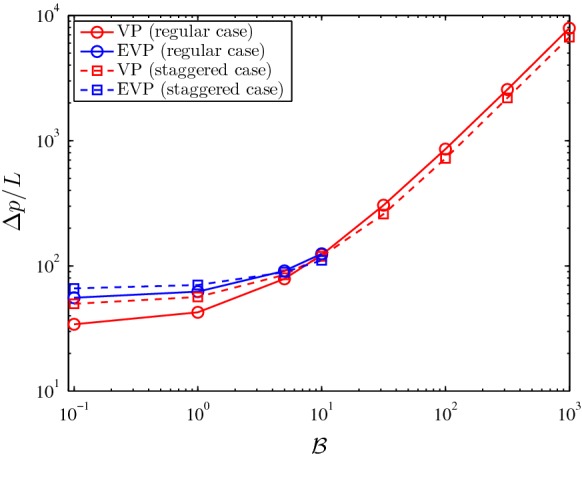
Pressure drop versus Bingham number. For the EVP cases the ${\mathcal{W}}i = 0.01$ and $\beta = 0.5$. The continuous lines with circles shows the computed pressure drop for the regular geometry and the discontinuous lines with squares are corresponding to the staggered geometry. Red and blue colors are used to distinguish between VP and EVP flows. (Color figure online)

We report $\varDelta p / L$ in Fig. 5 for a wide range of Bingham numbers. Considering either type of fluids, for small Bingham numbers, the pressure drop in the regular cases is smaller than for the staggered cases. On the other hand, Fig. 5 shows that the pressure drop in staggered cases is smaller than the regular cases for larger values of ${\mathcal{B}}$. We will investigate the limit of large Bingham numbers in detail in the next section.

An interesting feature is that, for a given Weissenberg number, we intuitively expect the pressure drop of an EVP flow to approach the value associated with the VP flow as we increase the Bingham number. This can be seen by considering that at fixed ${\mathcal{W}}i$ and increasing ${\mathcal{B}}$, the fluid will be more and more plastic rather than elastic. Indeed, this is confirmed in Fig. 5 where the gap between the pressure drop of the VP and EVP flows shrinks as we increase the Bingham number. Already at ${\mathcal{B}} \approx 10$, the difference in the pressure drop between VP and EVP flows is not detectable. This is corresponding to $\frac{{\mathcal{W}}i}{\mathcal{B}} \approx 0.001$. Here, we should briefly comment that for smaller values of $\frac{{\mathcal{W}}i}{\mathcal{B}}$, we did not reach the steady state in the EVP numerical solution (i.e., the flow was oscillatory), therefore, we do not present these results. However, a detailed discussion on this regime is given in Sect. 4.

At last we should comment that by changing $\beta$ from 0.1 to 0.9, no large variation in the pressure drop has been observed for EVP cases.

### ‘Flow/no flow’: limit of large $\mathcal{B}$

One of the most important questions in porous media applications of yield-stress fluids is the minimum pressure difference that should be imposed to ensure a continuous non-zero flow rate. In other words, for small values of the imposed $\varDelta \hat{p} / \hat{L}$, the flow cessation will happen with time. Only when the imposed pressure difference is above a certain value—the critical pressure drop—we expect the flow to start and remain continuous. For this aim, one can either attack the problem of finding the critical pressure drop by the present setting (see Sect. 2) or alternatively apply a constant pressure drop (i.e., $\varDelta \hat{p} / \hat{L}$) and compute the flow rate to see whether it is zero or non-zero. For the former approach, as mentioned in Sect. 2, we have scaled the velocity vector with the mean velocity at the inlet. Hence, the limit of ‘flow/no flow’ is equivalent to the limit of ${\mathcal{B}} \rightarrow \infty$. However, for the latter approach, the velocity scale is usually obtained by balancing the viscous stress with the pressure drop,

$$\begin{aligned} \hat{V} = \frac{\hat{R}^2}{\hat{\mu }} \frac{\varDelta \hat{p}}{\hat{L}}. \end{aligned}$$

Consequently, in this new setting, instead of the Bingham number, another non-dimensional number will describe the physics of the problem, i.e., the Oldroyd number,

$$\begin{aligned} {\mathcal{O}}d = \frac{\hat{\tau }_Y \hat{L}}{\hat{R} \varDelta \hat{p}}. \end{aligned}$$

Thus, finding the critical pressure gradient, $(\varDelta \hat{p} / \hat{L})_c$, is equivalent to calculating the critical Oldroyd number, ${\mathcal{O}}d_c$. For a VP flow it can be mathematically defined as,

5 $$\begin{aligned} {\mathcal{O}}d_c = \sup _{\varvec{v} \in {\mathcal{V}}, ~\varvec{v} \not = 0} \frac{\int _{\varOmega \setminus \bar{X}} \varvec{v}~\text{ d }A}{j(\varvec{v})} = \frac{\hat{\tau }_Y}{\hat{R} ~(\varDelta \hat{p} / \hat{L})_c}, \end{aligned}$$

using the variational analysis [38], where $\mathcal{V}$ is the set of admissible velocity test functions which are scaled with $\hat{V}$.

As mentioned above, in the present study we take the other approach so that finding the critical pressure drop and studying the limit of ‘flow/no flow’ is transformed to the limit of $\mathcal{B} \rightarrow \infty$. Indeed, when $\mathcal{B} \rightarrow \infty$, then the computed pressure drop goes to infinity as well, but the key parameter for calculating the critical Oldroyd number in the present approach is the following ratio,

6 $$\begin{aligned} {\mathcal{O}}d_c = \lim _{\mathcal{B} \rightarrow \infty } \frac{\int _{\varOmega \setminus \bar{X}} \varvec{u}~\text{ d }A}{j(\varvec{u})}. \end{aligned}$$

Here, we shall consider only the VP flow, since, as shown above, the EVP results are expected to converge to the VP ones for large enough Bingham numbers. Considering Eq. (4) in this limit (${\mathcal{B}} \rightarrow \infty$), we see that the plastic dissipation should balance the RHS, hence,

7 $$\begin{aligned} {\mathcal{B}} j(\varvec{u}) \sim {\mathcal{R}}e \frac{\varDelta p}{L} \int _{\varOmega \setminus \bar{X}} u~\text{ d }A . \end{aligned}$$

**Fig. 6:**
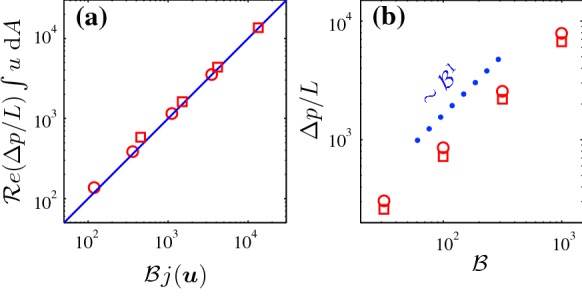
The red symbols are the computed data. Circles stand for the regular geometry and squares for the staggered case: **a** RHS of expression (4) versus the plastic dissipation. The blue line shows the one-one scaling; **b** pressure drop versus the Bingham number. (Color figure online)

In Fig. 6a, we plotted the numerical data to confirm the validity of this approximation. Moreover, in the limit of ${\mathcal{B}} \rightarrow \infty$, $j(\varvec{u})$ and $\int _{\varOmega \setminus \bar{X}} u~\text{ d }A$ reach their limiting values. Hence,

8 $$\begin{aligned} \frac{\varDelta p}{L} \sim {\mathcal{B}}. \end{aligned}$$

Figure 6b clearly show that this scaling is accurate in the limit of high $\mathcal{B}$.

To calculate the critical Oldroyd number, we should find the limiting values of the numerator and the denominator of the RHS of expression (6). Either it can be done using the numerical solutions or it can be estimated theoretically in some simpler cases. We will attempt to present a theoretical framework in what follows.

**Fig. 7:**
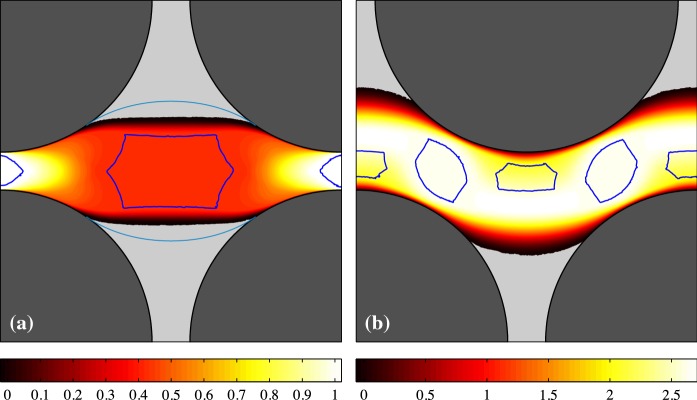
Contour of magnitude of the velocity, $\vert \varvec{u} \vert$, for the VP flow at $\mathcal{B}=1000$. The solid matrix is shaded in dark gray. Blue lines show the moving yield surfaces and static unyielded regions are filled with light gray. The green lines in panel **a** represents the last $\alpha$-lines in the slipline analysis. It can be seen that predicted $x^*$ from slipline analysis is in the good agreement with the AL code. (Color figure online)

The numerator can be estimated as *QL* where *Q* is the flow rate. Estimating $j(\varvec{u})$ theoretically, however, is not trivial and one needs some insights on the different physical mechanisms that contribute to the plastic dissipation in the flow. To provide some physical intuition, flows at very high Bingham number ($\mathcal{B}=1000$) are plotted in Fig. 7 for both considered geometries.

Roustaei et al. [38] have shown that there are three main contributions associated with the plastic dissipation in the intermediate size fractures with partial fouling. The case of regular geometry in the present study is falling into the same category, hence we can classify the main contributions to $j(\varvec{u})$ as follows:

1. In the wide part of the channel, there are static unyielded zones (light gray color in Fig. 7a). Between these static unyielded regions and the moving unyielded island (the border of which is painted in blue in Fig. 7a), there are two thin sheared boundary layers ($x^* \leqslant x \leqslant L - x^*$) in which plastic dissipation is significant.
2. There are pseudo-plug regions ($0 \leqslant x \leqslant x^*$ & $L - x^* \leqslant x \leqslant L$) in which the bulk flow is expanding (or contracting) because of the axial variation of the solid surface and hence there is another major contribution to the plastic dissipation in these regions.
3. The third non-negligible contribution is coming from the thin layers of fluid which are being sheared adjacent to the solid walls at the entrance and exit parts of the channel.

In a coordinate system with the origin at the center of the inlet gap, exploiting the symmetry of the flow in the axial direction (due to the low inertia), $j(\varvec{u})$ takes the form,

9 $$\begin{aligned} j(\varvec{u}) = \frac{\pi }{2} \log \left[ 1+8h(x^*)\right] +\frac{4~x^*}{1+8h(x^*)}+\int _{0}^{x^*} \frac{4~\text{ d }x}{1+8h} \end{aligned}$$

where, *h* is the distance of the solid surface from the centerline of the domain, i.e.,

$$\begin{aligned} h=1.125-\sqrt{1-x^2}. \end{aligned}$$

For the detailed derivation of expression (9), readers are referred to [38]. Please note that $x^*$ is the position of the starting point of the static unyielded region on the surface of the solid topology. Numerical computations (AL code) gives $j(\varvec{u}) \approx 3.47$, while using expression (9) with $x^*$ extracted from the numerical result, the calculated $j(\varvec{u})$ is approximately 3.57 which is a very close estimation. These deduce ${\mathcal{O}}d_c \approx 0.13$ for the regular geometry.

The single issue which is remained to be addressed in this theoretical framework is finding $x^*$ independent of the full numerical solution (AL code) of the problem. To do this theoretically, we can rely on the slipline analysis (which is a powerful tool in the regime of 2D perfectly-plastic flows [4]). Sliplines are the characteristic lines of the hyperbolic set of equations which governs the 2D perfectly-plastic flows and physically they show the direction of the maximum shear stress in the domain (i.e., the directions in which the shear stress is equal to $\pm {\mathcal{B}}$). This method, in the context of viscoplastic fluid mechanics, has recently opened an avenue for studying the yield limit flows. For details on how to use the slipline analysis and the calculation procedure, readers are referred to Refs. [5, 7]. We have adopted the same method in the present study. Here we assume that the maximum shear stress will occur on the solid surfaces. Therefore, sliplines are tangent to the solid topologies. Since the shear stress is zero on the horizontal axis of the channel (due to symmetry), sliplines should make $\pm \pi /4$ angle with $y=0$ line. Moreover, sliplines should be orthogonal/tangent to the vertical symmetry ($x=1.125$ line) if they touch it.

**Fig. 8:**
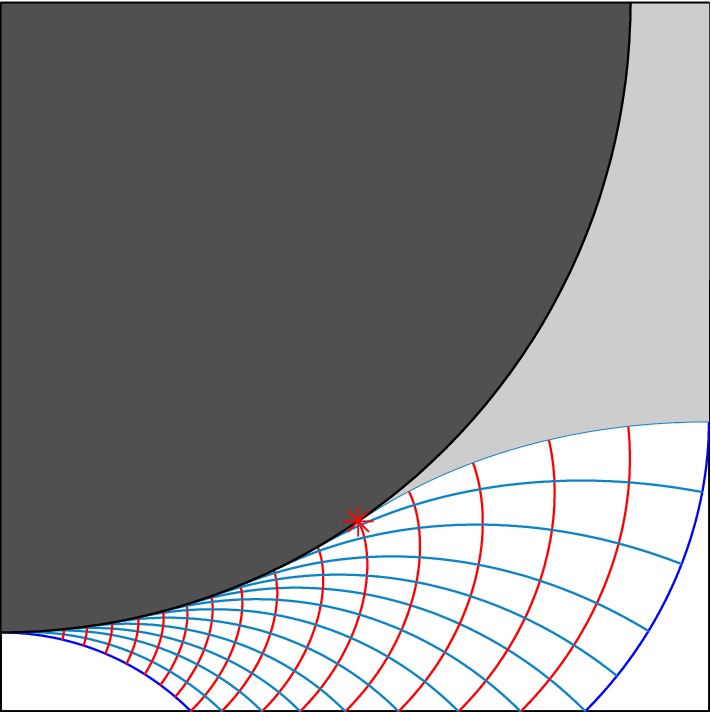
Sliplines for the regular geometry case (left top quarter of the $\varOmega$). Only a selection of sliplines is plotted to avoid smearing the figure. Two yield surfaces are shown in blue. Green lines are the $\alpha$-lines while the $\beta$-lines are illustrated in red. The red asterisk shows the start position of the static unyielded zone. Putting the center of the coordinate system at the center of the inlet gap, $x^* \approx 0.57$. (Color figure online)

The generated sliplines for the present problem (the regular geometry) are shown in Fig. 8. Although yield surfaces predicted by slipline analysis are not perfectly matched to the ones for the viscoplastic flow (Fig. 7a), qualitatively, same behavior of flow can be observed. On the other hand, the value of $x^*$ obtained from the slipline approach ($\approx 0.57$) is very close to the one from the AL code ($\approx 0.59$). It should be noted that this small difference is most likely due to the fact that the VP simulation has been performed at a finite, yet large, Bingham number (${\mathcal{B}}=1000$). Of course, larger values of the Bingham number will increase the unyielded regions, so that, $x^*$ will take slightly smaller values (i.e., the computed $x^*$ will be closer to the slipline estimation).

For the the flow in the staggered geometry used in the present study, estimating different contributions to $j(\varvec{u})$ within the presented theoretical framework is not trivial due to the “affine” self-selection pattern of the flow. However, one can still use expression (6) to extract the critical Oldroyd number from the numerical solution. For the staggered case, $\mathcal{O}d_c$ will be approximately 0.0021.

To conclude, we have formulated a framework for estimating the critical pressure drop which is required to ensure the flow of a yield-stress fluid inside the model porous media. We have presented a simple analytical method (expression (9)) to estimate the critical pressure drop in the regular geometry. To use this method, one only needs to know the location of the starting point of the fouling layers at high Bingham numbers. This has been obtained by slipline analysis, which does not require the computations of the flow field of a yield-stress fluid.

## Summary and discussion

In this paper, we have studied the relevance of VP and EVP flows, in the context of flows in porous media. Two structures of the solid topology have been considered: regular and staggered sets of cylinders.

We have kept our attention to EVP flows at a small Weissenberg number to compare the features with the corresponding VP flows. Generally same behaviors can be observed between VP and EVP flows in the model geometries at small Weissenberg number with a small difference in the unyielded zones: larger unyielded islands are moving in the middle of the EVP flow compared to the VP flow, while the static unyielded zones are more or less the same. The main reason of this difference is the different stress field solutions, however, velocity fields are quite comparable. It should be noted that unyielded zones in the VP and EVP problems are physically different since the unyielded zones in an EVP flow are in fact ‘viscoelastic solids’ which can have non-zero strain rates.

We have shown that as $\frac{{\mathcal{W}}i}{\mathcal{B}} \rightarrow 0$, the difference in the pressure drops of VP and EVP flows will be smaller and smaller: approximately at $\frac{\mathcal{W}i}{\mathcal{B}} \approx 0.001$, the difference becomes negligible. However, this does not contain any information about the convergence behavior of VP and EVP flows in general. Indeed, although the pressure drops and the velocity fields may be comparable, still the stress fields could be quite different. In general, for any considered physical problem, we intuitively expect that VP and EVP solutions converge together as $\frac{{\mathcal{W}}i}{\mathcal{B}^n} \rightarrow 0$; where *n* is the potential power which depends on the physics of the problem. For instance, in the context of Stokes EVP flow over an obstacle, Cheddadi et al. [9] split this regime into three sub-regimes:

1. At high $\mathcal{W}i$ numbers (typically of *O*(1)), we expect non-linearities from the elastic regime and so VP and EVP flows are different.
2. At moderate $\mathcal{W}i$ numbers (larger than $10^{-2}$ but less than 1), the exact value of the Weissenberg number does not affect the flow significantly.
3. At very small Weissenberg numbers, the fore-aft asymmetry in the flow will dramatically increase (it should be mentioned that the Stokes VP flow over an obstacle is perfectly symmetric).

However, we expect by putting $\mathcal{W}i=0$ in any EVP flow, the solution will be the same as the VP flow. This suggests that a fourth regime will emerge at an *ultra* small Weissenberg number, in which the difference between the VP and EVP solutions shrinks (e.g., fore-aft asymmetry will disappear in the Cheddadi et al. [9] problem). Otherwise, it means that the transition from VP to EVP flows is discontinuous, which is unintuitive. In the present study, we have observed that for values of Bingham number more than 10 (provided that $\mathcal{W}i$ is fixed at 0.01), the EVP flow will be unsteady and some oscillations in the pressure drop and yield surfaces will occur during the course of the flow. It has been previously observed as well in different physical problems (e.g., see Ref. [30]). The origin of these oscillations could be either numerical or rheological. Hence, we did not report the non-steady cases in the present study. We shall leave the study of this interesting limit (i.e., *ultra* small values of $\frac{{\mathcal{W}}i}{\mathcal{B}}$) to the future works in which one may use a different numerical method and/or more complex rheological EVP model.

Possible scalings have been proposed in the limit of $\mathcal{B} \rightarrow \infty$ which is of great importance practically. Most interestingly, here we have presented a method to calculate the critical pressure difference in the limit of ‘flow/no flow’ in the porous media. This critical pressure difference is vital for ensuring a continuous flow in the system. For the regular geometry, a theoretical model has been proposed to calculate plastic dissipation which can be used to calculate the critical pressure difference or in non-dimensional space, the critical Oldroyd number as defined above. We have also shown that slipline analysis is useful in this problem as it allows us to track the static unyielded zones and use them in the theoretical model. The next challenge would be identifying the different sources of plastic dissipation (in the limit of $\mathcal{B} \rightarrow \infty$) also for more complex configurations, such as the staggered arrangement used here.

## Figures and Tables

**Fig. 9:**
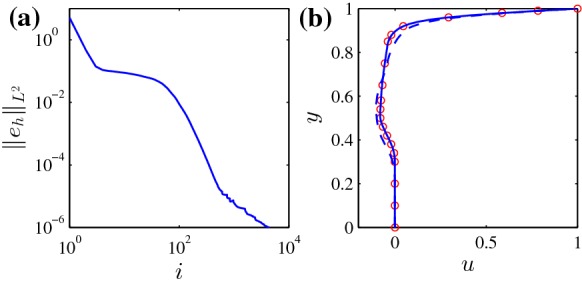
Performance of the AL code and mesh adaptation for a lid-driven cavity flow (${\mathcal{R}}e=1000, {\mathcal{B}}=10$):  **a** residual convergence versus the number of iterations,  **b** horizontal component of velocity at $x=0.5$ for the whole height of the cavity; the red symbols are the data from Ref. [43]; discontinuous line shows the present computation with quasi-uniform mesh, and continuous line is associated with the adapted mesh. (Color figure online)

## Appendix: Numerical details

In the current study, we use two different methods for solving the flow of VP and EVP fluids. A detailed explanation of the method used for the EVP problem has been recently published by Izbassarov et al. [28]. For a complete clarification of the method for the porous media flow, readers are referred to Ref. [16]. To highlight, for the EVP flow a 3D numerical solver is used that combines highly scalable FFT-based pressure solver with the evolution equation for non-Newtonian stresses. The volume penalization Immersed Boundary Method (IBM) [20] is used to generate the complex geometries. The log-conformation method [21, 27] is used to overcome the well-known high Weissenberg number issue. The equations of motion are solved on a regular Cartesian uniform grid. Time marching is performed with a third-order explicit Runge–Kutta scheme except for the extra stress which is advanced with the Crank–Nicolson method. The spatial derivatives are approximated with second-order centered finite differences except for the advection term in the constitutive equation where the fifth-order WENO [41] is adopted.

For the VP problem, however, due to the discontinuous nature of the Bingham fluid, classical methods cannot be used. The Augmented Lagrangian method (AL), on the other hand, is a well-known robust method for solving the equations associated with these fluids; this is based on convex optimization techniques and converts the problem to finding the saddle-point of the *minimum* and *maximum* principles (see Refs. [24, 32, 34]). We use the same method in the present study to solve the VP problem. Regarding the discretization of the inertial term on the left hand side of Eq. (1), the characteristic method [3] has been employed as implemented by Roustaei and Frigaard [36]. This method consists of a pseudo-time stepping towards the steady-state solution of the inertial problem. The convergence criterion has been set to,

$$\begin{aligned} \text{ max } \left( \Vert \varvec{u}^{n+1} - \varvec{u}^n \Vert _{L^2},~ \Vert \varvec{\gamma }^{n+1} - \dot{\varvec{\gamma }}(\varvec{u}^{n+1}) \Vert _{L^2} \right) \geqslant 10^{-7} \varDelta t, \end{aligned}$$

where superscripts represents the number of iterations, $\varvec{\gamma }$ the relaxed strain rate in the augmented Lagrangian method, and $\varDelta t$ is the pseudo-timestep.

Anisotropic adaptive mesh is used to simulate the VP problem. Regarding the adaptation method, our approach is the same as Ref. [34], and has been validated in Ref. [6]. Finally, it should be mentioned that all the above steps (for the VP problem) have been implemented in an open-source C++ finite element environment, FreeFEM++ [25]. To validate the whole implementation of the VP problem, the performance of the code is examined for a $1 \times 1$ inertial lid-driven cavity flow in Fig. 9.
